# Low Prevalence of *Pfcrt* Resistance Alleles among Patients with Uncomplicated Falciparum Malaria in Niger Six Years after Chloroquine Withdrawal

**DOI:** 10.1155/2014/614190

**Published:** 2014-11-23

**Authors:** Adamou Salissou, Halima Zamanka, Brigitte Biyghe Binze, Taiana Rivière, Magalie Tichit, Maman Laminou Ibrahim, Thierry Fandeur

**Affiliations:** ^1^Université de Ouagadougou, BP 364, Ouagadougou, Burkina Faso; ^2^Unité de Parasitologie, Centre de Recherche Médicale et Sanitaire, 634 Bd de la Nation, BP 10887, YN034, Niamey, Niger; ^3^Unité de Parasitologie Médicale, Centre International de Recherches Médicales de Franceville, BP 769, Franceville, Gabon; ^4^Genopôle de l'Ile de France, Plate-Forme Génomique, Institut Pasteur, 28 Rue du Dr ROUX, 75015 Paris, France; ^5^Division Internationale, Institut Pasteur, 28 Rue du Dr ROUX, 75015 Paris, France

## Abstract

Chloroquine (CQ) resistance is widespread in Africa, but few data are available for Niger. *Pfcrt* haplotypes (aa 56–118) and *ex vivo* responses to CQ and amodiaquine were characterized for 26 isolates collected in South Niger from children under 15 years of age suffering from uncomplicated *falciparum* malaria, six years after the introduction of artemisinin-based combinations and the withdrawal of CQ. The wild-type *Pfcrt* haplotype CVMNK was found in 22 of the 26 isolates, with CVIET sequences observed in only three of the samples. We also describe for the first time a new CVINT haplotype. The *ex vivo* responses were better for CVMNK than for CVIET parasites. *Pfcrt* sequence data were compared with those obtained for 26 additional parasitized blood samples collected in Gabon, from an area of CQ resistance used as a control. Our findings suggest that there has been a significant decline in CQ-resistant genotypes since the previous estimates for Niger were obtained. No such decline in molecular resistance to CQ was observed in the subset of samples collected in similar conditions from Gabon. These results have important implications for public health and support the policy implemented in Niger since 2005, which aims to increase the efficacy and availability of antimalarial drugs whilst controlling the spread of resistance.

## 1. Introduction

Malaria remains the principal endemic disease and the leading cause of morbidity and mortality in Niger. Despite the strengthening of control measures and the complementary nature of the interventions implemented, the number of cases has steadily increased, tripling over the last 20 years, from 1,162,824 in 1990 to 3,641,003 in 2010 and 3,525,112 in 2012 [1]. The reasons for the deterioration of the malaria situation in Niger have not been fully elucidated but are probably multifactorial. The weakening of malaria control programs, the poor treatment-seeking behavior, and the improved reporting of suspected cases are certainly major factors that have contributed to some extent to the apparent worsening of the malaria burden in Niger. At the same time, it has been suggested that a reduced effectiveness of first-line antimalarials has greatly contributed to malaria resurgence in parts of Africa. In Niger, however, the contribution of the spread of resistance to malaria trends observed over recent years is mostly unknown due to the shortage of data [2, 3]. The first cases of resistance to chloroquine (CQ) were reported in Niger at the beginning of the 1990s [4]. The rare tests of therapeutic efficacy carried out since, in 1998 and 2001, have confirmed the circulation of CQ-resistant (CQR) parasites in Niamey, but with only a moderate treatment failure rate (<25% in all cases, before PCR correction) [5, 6]. The trials carried out with the sulfadoxine-pyrimethamine combination (SP) at the sentinel sites of the National Malaria Control Program (NMCP) in Niger also reported reasonably high sensitivity, with an adequate clinical and parasitological response in 78% of cases (Dr. A Salissou, NMCP Niger, personal communication). In 2005, the health authorities of Niger introduced the use of artemether-lumefantrine (AM-LM) combinations as a first-line treatment for malaria. The artesunate-amodiaquine (AS-AQ) combination was then recommended in 2008. These artemisinin-based combination treatments (ACTs) are still in use as first line treatment. Niger is also one of the few countries to have benefited from the Affordable Medicine Facility-malaria (AMFm) program of the Global Fund since 2011 [7]. These interventions led to the implementation of a resistance monitoring system, to check the efficacy of ACTs, and of other more conventional antimalarial drugs, including SP, AQ, and CQ. The use of CQ has been officially prohibited in Niger since 2006, and changes in the efficacy of this antimalarial drug over time are therefore a good indicator of the performance of the national policy for malaria control and infection management [8]. However, too few data are available for the resistance situation in Niger to be evaluated with the required level of precision. Efforts have been made to bridge this gap through tests of treatment efficacy for AM-LM and AS-AQ combinations. In a recent study, carried out in 2011 in southern Niger, we found that AM-LM and AS-AQ combinations were satisfactory for the treatment of uncomplicated* P. falciparum* malaria, with adequate clinical and parasitological responses obtained in 94.8% and 97.1% of cases, respectively, after PCR correction (Ibrahim ML et al., in preparation). This work was coupled with the* ex vivo* assessment of parasite responses to a panel of antimalarial drugs and genotyping for* Pfcrt*-K76T by PCR-RFLP. Consistent with previous reports, similar trends were observed* in vitro* with artemisinin derivatives and other partner molecules, and also, more surprisingly, with CQ and AQ, with 93% and 96% of isolates responding adequately* in vitro* to these drugs, respectively. Only 10% of isolates displayed a mutated codon at position 76 [9].

In this study, we aimed to confirm the unusual good sensitivity of* P. falciparum* parasites to amino-4-quinoleine for an additional subset of parasitized blood sample from Niger and to investigate the correlation between* ex vivo* responses to CQ and AQ, and a polymorphism at the* Pfcrt* locus, by the sequencing of PCR products [10, 11]. Sequence data from Niger were compared with those obtained for parasitized blood samples collected from an area of CQ resistance in Gabon used as a control.

## 2. Patients and Methods

### 2.1. Parasitized Blood Samples

Parasitized blood samples were obtained from febrile children suffering from uncomplicated* falciparum* malaria recruited for a therapeutic efficacy test carried out in 2011 at the Gaya Health Center in the Dosso region, 250 km south of Niamey (3.44°N, 11.9°E). The study protocol was approved by the National Ethics Committee of the Nigerien Ministry of Health. A small subset of these samples was analyzed in a previous study and the results obtained were presented in a preliminary report [9]. In this study, we focused on another subset of the samples collected as a part of this* in vivo* test at the Gaya site that had not previously been studied. The samples analyzed were selected on the basis of the* in vitro* data that were available at the time of the study. Another set of isolates from Gabon was used as a control group. These samples were collected in 2013 from febrile outpatients attending the A. Bongo Referral Hospital in Franceville (1°38′S and 13°35′E) located in southeast Gabon in the Haut Ogooué province. The study protocol was approved by the National Ethics Committee of Gabon (decision 0015, February 4, 2013).* Ex vivo* tests of sensitivity to antimalarial drugs were carried out only for the Nigerien samples. In all cases, informed written consent was obtained before sampling and data were collected and processed anonymously.

### 2.2. *Ex Vivo* Studies and* Pfcrt *Genotyping

The* ex vivo* susceptibilities of* falciparum* isolates to CQ and AQ were assessed only for the Nigerien samples, in a classical 48 h isotopic test, as fully described elsewhere. Briefly, fresh isolates were examined shortly after blood collection in microplates that had been coated with serial dilutions of CQ diphosphate and of AQ dihydrochloride dihydrate (Sigma C6628 and A2779, resp.). Parasite growth was assessed by measuring the incorporation of [^3^H]-hypoxanthine (0.5 *μ*Ci/well), as previously described [12]. The results of the assay are expressed as the 50% inhibitory concentration (IC_50_) defined as the concentration at which 50% of [^3^H]-hypoxanthine incorporation was inhibited, with respect to the values obtained for the drug-free control wells. Parasite growth and IC_50_ values were determined by log probit approximation. The suitability of the test plates for* in vitro *testing was monitored with the reference 3D7 Africa and W2 Indochina strains, maintained in continuous culture and presenting known responses to the drugs tested. These reference parasite lines were obtained from J Lebras (centre national de référence du paludisme, Paris). The* Pfcrt* haplotypes of Nigerien and Gabonese isolates were determined by a PCR sequencing approach. Genomic DNA was extracted (QIAamp DNA blood kit, QIAGEN) using 200 *μ*L of parasitized blood samples according to manufacturer recommendations and from cultured 3D7 and W2 parasites for the control.* Pfcrt* sequences (a 280 bp fragment) were amplified at the CERMES and CIRMF facilities by conventional PCR, with the forward primer 5′CGACCTTAACAGATGGCTCA3′ and the reverse primer 5′TTTGAATTTCCCTTTTTATTTCCA3′. Briefly, amplifications were performed in 50 *μ*L final reaction volume containing DNA template, 1 *μ*M each primer, 200 *μ*M each dNTP, 2 mM MgCl_2_, and 2.5 U Taq polymerase (*GoTaq* Flexi DNA Polymerase, Promega). The PCR amplification conditions were 1 cycle of denaturation (95°C for 2 min., 56°C for 1 min., 72°C for 1 min.) followed by 5 cycles (95°C for 30 sec., 56°C for 1 min., 72°C for 1 min) and 33 cycles (95°C for 10 sec., 55°C for 1 min., 72°C for 1 min.) and an additional extension cycle (95°C for 10 sec., 55°C for 1 min., 72°C for 10 min.). The PCR products were cleaned using QIAquick PCR Purification Kit (QIAGEN) and transported to the Institut Pasteur Genomics (Paris, France) Platform or to Biofidal Laboratories (Villeurbanne, France) for sequencing.

## 3. Results and Discussion

**Figure 1:**
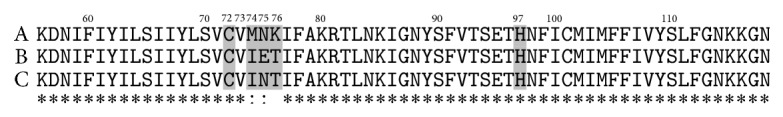
*Pfcrt* (aa 56–118) haplotypes observed in isolates from Niger and Gabon (XM_001348968). The three haplotypes identified differ by amino acids found at positions 74–76, MNK (A), IET (B), and INT (C). Gray shading indicates residues that are known to be polymorphic.

Mean [range] parasitemia (% infected red blood cells) at admission was 6.4 [1.5–35.0] in isolates from Niger, whereas the samples obtained from Gabonese febrile individuals displayed lower parasite densities 0.95 [0.02–3.95]. Three haplotypes were identified in these isolates (Figure 1). The CVMNK (A) wild-type haplotype (aa 72–76) was found in 22 of the 26 (85%) Nigerien isolates and in only 7 out of the 26 (27%) Gabonese samples examined. The CQ-susceptible 3D7 reference strain contains the CVMNK haplotype. The CVMNK sequence has been consistently reported in all CQ-susceptible isolates collected to date, regardless of geographic origin. The H97L/Q polymorphism previously observed in Asia, in South America, and in Niger about 10 years ago was not detected in this subset of samples [13, 14]. The triple-mutant CVIET (B) haplotype was present in 11% (3/26) of the Nigerien and in 73% (19/26) of Gabonese samples. The CQ-resistant W2 reference strain contains the CVIET haplotype. The CVIET haplotype, which is classically associated with CQR parasites, is widespread throughout Africa. Finally, the double-mutant CVINT haplotype (C) was found in a single isolate of Nigerien origin (4%) among the samples analyzed (Table 1) [13]. The existence of the CVINT haplotype was predicted by evolutionary analyses of* Pfcrt* sequences, but this is the first time that this form has been unequivocally detected (infection with a single isolate presenting a single genotype on typing with* Pfmsp1* and* Pfmsp2*) in conditions of natural transmission [15]. The digestion of* Pfcrt* fragments with* Apo-*I and the microarray technique used for routine screening of the* Pfcrt*76T mutation cannot distinguish between the CVIET and CVINT sequences. This may have contributed to the late detection of this haplotype in field samples. This study, although limited, is the first to describe* Pfcrt* haplotypes at codon 56–118 in isolates from Niger.

The* ex vivo *responses to CQ of the parasites from Niger were significantly (*P* < 0.05 Student's *t*-test) better for CVMNK parasites (13.9 nM) than for CVIET parasites (132.5 nM). By contrast, the difference in the mean IC_50_ of AQ between CVMNK (15.8 nM) and CVIET parasites (20.2 nM) was not significant (*P* = 0.33, Student's *t*-test). The isolate of haplotype CVINT responded least well to AQ, with an IC_50_ of 46.7 nM, which is close to the* in vitro* limit of susceptibility to AQ (AQS of <60 nM), whereas the response to CQ was satisfactory, with an IC_50_ of 24.4 nM, below the threshold for* in vitro *resistance (CQR of >100 nM) [16]. The malaria management policy in Niger has changed substantially in recent years, with the gradual replacement of CQ and SP with ACTs, some of which include AQ in their formulation, potentially accounting for changes in* Pfcrt* sequences in circulating isolates and favoring the selection of new mutations. Both CQ and AQ belong to the 4-aminoquinoline class of drugs, but cross-resistance between them is moderate, suggesting that the mechanisms of resistance to these molecules are linked, but different [17].

Resistance to CQ is widespread in Africa, but unevenly distributed between regions. Failure rates are high in Congo (95.7%), Mali (90.5%), and Nigeria (58.5%) and lower in Burkina Faso (between 6.6 and 25%) [18–22].* In vivo* data for Niger and Gabon are scarce and those available are now very old. Determinations of the frequency of the* Pfcrt*T76 mutation can be used to estimate the level of resistance to CQ and its variation over time. This makes it possible to address the issue of resistance to CQ without having to carry out therapeutic efficacy tests, which are laborious and expensive to perform, and raise ethical issues. The ratio of the frequency of the* Pfcrt*T76 mutation to the prevalence of parasitological or clinical treatment failure defines a genotypic resistance index (GRI) or a genotypic clinical failure index (GFI) [23]. These indices are very similar in regions with similar epidemiological contexts. The GFIs reported for the neighboring countries, Mali, Burkina Faso, and Ghana, differ by a factor of one to three [21–25]. By applying these values to the prevalence of 15% observed for the* Pfcrt*76T mutation in this collection of samples from Niger, the predicted rate of clinical failure with CQ would currently be between 5 and 15%. These values are similar to or better than those reported for comparable zones of transmission in Burkina Faso, in which the mean frequencies of treatment failure associated with the* Pfcrt*76T mutation calculated in 1998 and 2003 were of 25 and 50%, respectively [21]. PCR-RFLP studies carried out in Niger between 2001 and 2011 showed fluctuations in the frequency of the* Pfcrt*76T mutation. The proportions of isolates from Niamey, carrying the mutation were 26.9% and 45.5% in 2001 and 2003, respectively [26, 27]. Niamey, the capital of Niger (13°31′N and 2°06′E) has a semiarid climate, with a rainy season lasting about five months from June to October during which malaria transmission is sustained. The mean frequency calculated for several sites in Niger was 50.8% for 2005-2006 at various sites along valley of the Niger river and that calculated for Banizoumbou and Zindarou was 20% in 2003 and 44% in 2006 [14, 27]. Zindarou (13°26.09N/2°55.23E) and Banizoumbou (13°32.03N/2°39.66E) are two villages of comparable size (resp. 500 and 1,000 inhabitants), 70 km far from Niamey and 20 km distant from each other. These values should be compared with the values of 11% (PCR-RFLP, [9]) and 15% (direct sequencing, this study) obtained at the Gaya site in 2011, after the introduction of ACTs. This study therefore confirms the low level of resistance to CQ* in vitro* and the low prevalence of the* Pfcrt*K76T mutation in southern Niger. A comparison of our data with those collected earlier in Niger between 2001 and 2006 indicated that the frequency of CQR genotypes had significantly decreased: the mean frequency of such genotypes fell from 37.4% for 2001–2006 to about 13% in 2011 (*χ* ^2^ = 5.4; *P* = 0.0002). This decrease was accompanied by an increase in the frequency of the CQ-susceptible* Pfcrt* CVMNK genotype and the emergence of a new genotype,* Pfcrt* CVINT, possibly selected under the pressure of the new treatments combining artesunate and AQ introduced into Niger in 2008. The total number of cycles applied (40), the size of the amplified fragment (<300 bp), the electropherogram pattern and the error rate of our Taq polymerase (about 10^−5^) all suggest that the possibility of the misincorporation of two incorrect nucleotides at either codon 75 (CVIET > CVINT), or codons 74 and 76 (CVMNK > CVINT) is very low. The possibility that amplification of the CVINT allele from CVMNK or CVIET sequences (and thus that detection of a CVINT allele) resulted from a PCR artefact is unlikely. We therefore are confident with the result and feel that CVINT sequence represents a* bona fide* new* Pfcrt* allele. The frequency and distribution of this new haplotype should be monitored. Another indicator of the profound change in the drug pressure exerted on the parasite populations of Niger in recent years is the finding that the* Pfcrt*97Q mutation, which was present at a frequency of 21.4% in a region only 150 km north of Gaya in 2006, was not detected in samples collected in 2011 [14]. Overall, these results suggest a gradual inversion of the pattern of CQR genotypes and phenotypes in Niger following the withdrawal of CQ and the introduction of ACTs in this country.

No such reversal of sensitivity to CQ has yet been observed in Gabon, where levels of resistance to conventional antimalarial drugs remain high. The rate of CQR is about 50% higher than that in neighboring countries, and an* in vivo* assay of therapeutic efficacy conducted in 2001 on Gabonese children reported a treatment failure rate of 100% at day 28 [28]. Similarly, molecular studies have demonstrated the fixation of the mutated* Pfcrt* allele, with more than 97% of isolates carrying the K76T mutation [29]. This study, conducted on a small subset of samples collected in Franceville, shows a very similar level genotypic resistance to CQ with 73% of isolates harboring CVIET sequences. These results validate the molecular data reported for isolates from Niger and indicate that several years after the withdrawal of CQ as a first-line treatment in Gabon, the prevalence of the mutant* pfcrt *allele remains extremely high. This finding, thus, stands in contrast to a more pronounced development towards CQ sensitivity in Niger. The reasons for these differences in CQ resistance between Gabon and Niger remain unclear, particularly as transmission rates would be expected to be higher in Gabon (latitude 1°38′S) than in Niger (latitude 11°90N), with perennial transmission and entomological inoculation rates of 0.05–0.74 in Niger and 0.23–0.76 in Gabon [30, 31]. It has been suggested that the use of combinations of artesunate and AQ may have resulted in the continuing selection of the mutant* pfcrt* haplotype, even after the withdrawal of CQ in Gabon [29]. However, Niger, which switched the use of conventional antimalarial drugs for ACT under similar conditions, has seen its level of resistance to CQ continues to decline to a very low level. This element alone is therefore insufficient to account for the different outcomes. Instead, the implementation of the Affordable Medicines Facility mechanism in Niger may have helped to improve the malaria situation over recent years, by preventing the spread of drug resistance, in particular.

Increases in morbidity and mortality due to malaria have been associated with the emergence and propagation of resistance to antimalarial drugs. In Niger, at least in the southern part of the country, the parasites examined had an adequate response to artemisinin derivatives and conventional antimalarial agents, and no direct link could be establish between resistance and an increase in malaria incidence. It should be noted that mortality has fallen, from 0.21 in 2000 to 0.06 in 2010, indicating that the interventions carried out as part of the NMCP of Niger have not been without effect but instead that their impact is difficult to evaluate due to confounding factors. The improvements in the national health cover provided by health structures, the introduction in 2006 of free healthcare for children under the age of five years, and the rise of the population (particularly for the under-fives) have led to an increase in healthcare facility attendance in recent years and, thus, to a technical augmentation in the number of cases reported. Furthermore, in the absence of biological case confirmation, the contribution of malaria to fever statistics is largely overestimated in Niger where transmission rates are moderate [32]. This highlights the absolute necessity of confirming malaria cases in Niger by a biological test, before notification and treatment, and of the long-term surveillance of antimalarial drug resistance.

These findings validate the treatment policy implemented by the Ministry of Health in Niger, with the support of the Global Fund. However, the study included only a small number of isolates, possibly too few for the establishment of a definitive link between these changes and a return to greater susceptibility to CQ. Additional studies in other areas of Niger with different epidemiological contexts are required to determine whether the observations made at Gaya can be extended to the whole of Niger.

## Figures and Tables

**Table 1:** Relationship between genotype at the *Pfcrt* locus and response *ex vivo* to chloroquine and amodiaquine.

*Pfcrt* genotype	NIGER						GABON	
	*n*	Mean parasitemia [range] %	CQ geometric mean IC_50_ [95% CI] nM		AQ geometric mean IC_50_ [95% CI] nM		*n*	Mean parasitemia [range] %
A (CVMNK)	22	5.9 [1.5–35]	13.9 [10.2–17.2]	*P* = 0.03	15.8 [12.8–18.8]	*P* = 0.33	7	0.86 [0.02–2.22]
B (CVIET)	3	11.3 [2–25]	132.5 [112.5–152.5]		20.2 [9.4–31.0]		19	0.97 [0.02–3.95]
C (CVINT)	1	2.0	24.4		46.7		0	—

## References

[1] Ministère de la santé du Niger/OMS http://www.snis.cermes.net/donnees.php.

[2] White N. J., Nosten F., Looareesuwan S. (1999). Averting a malaria disaster. *The Lancet*.

[3] Cohen J. M., Smith D. L., Cotter C., Ward A., Yamey G., Sabot O. J., Moonen B. (2012). Malaria resurgence: a systematic review and assessment of its causes. *Malaria Journal*.

[4] Gay F., Diquet B., Katlama C., Fassin D., Turk P., Datry A., Danis M., Gentilini M. (1991). Report of chloroquine resistance malaria in Niger. *Therapie*.

[5] Dugelay F., Adehossi E., Adamou S., Ousmane I., Parzy D., Delmont J., Parola P. (2003). Efficacy of chloroquine in the treatment of uncomplicated, *Plasmodium falciparum* malaria in Niamey, Niger, in 2001. *Annals of Tropical Medicine and Parasitology*.

[6] Parola P., Ali I., Djermakoye F., Crassard N., Bendavid C., Faugère B., Condomines P. (1999). Chloroquine sensitivity of *Plasmodium falciparum* at the Gamkalley Clinic and the Nigerian armed forces PMI (Niamey, Niger). *Bulletin de la Société de Pathologie Exotique*.

[7] The Global Fund Final Report of the Independent Evaluation of AMFm Phase 1. http://www.theglobalfund.org/en/privatesectorcopayment/amfmindependentevaluation/.

[8] Laufer M. K., Takala-Harrison S., Dzinjalamala F. K., Stine O. C., Taylor T. E., Plowe C. V. (2010). Return of chloroquine-susceptible falciparum malaria in malawi was a reexpansion of diverse susceptible parasites. *The Journal of Infectious Diseases*.

[9] Issaka M., Salissou A., Arzika I., Guillebaud J., Maazou A., Specht S., Zamanka H., Fandeur T. (2013). *Ex Vivo* responses of *Plasmodium falciparum* clinical isolates to conventional and new antimalarial drugs in niger. *Antimicrobial Agents and Chemotherapy*.

[10] Djimdé A., Doumbo O. K., Cortese J. F., Kayentao K., Doumbo S., Diourté Y., Dicko A., Su X.-Z., Nomura T., Fidock D. A., Wellems T. E., Plowe C. V., Coulibaly D. (2001). A molecular marker for chloroquine-resistant falciparum malaria. *The New England Journal of Medicine*.

[11] Happi C. T., Gbotosho G. O., Folarin O. A., Sowunmi A., Bolaji O. M., Fateye B. A., Kyle D. E., Milhous W., Wirth D. F., Oduola A. M. J. (2006). Linkage disequilibrium between two distinct loci in chromosomes 5 and 7 of *Plasmodium falciparum* and *in vivo* chloroquine resistance in Southwest Nigeria. *Parasitology Research*.

[12] Lim P., Chim P., Sem R., Nemh S., Poravuth Y., Lim C., Seila S., Tsuyuoka R., Denis M. B., Socheat D., Fandeur T. (2005). *In vitro* monitoring of *Plasmodium falciparum* susceptibility to artesunate, mefloquine, quinine and chloroquine in Cambodia: 2001-2002. *Acta Tropica*.

[13] Ecker A., Lehane A. M., Clain J., Fidock D. A. (2012). PfCRT and its role in antimalarial drug resistance. *Trends in Parasitology*.

[14] Ibrahim M. L., Steenkeste N., Khim N., Adam H. H., Konaté L., Coppée J.-Y., Ariey F., Duchemin J.-B. (2009). Field-based evidence of fast and global increase of *Plasmodium falciparum* drug-resistance by DNA-microarrays and PCR/RFLP in Niger. *Malaria Journal*.

[15] Awasthi G., Satya G. B. K., Das A. (2012). *Pfcrt* haplotypes and the evolutionary history of chloroquine-resistant *Plasmodium falciparum*. *Memórias do Instituto Oswaldo Cruz*.

[16] Pradines B., Hovette P., Fusai T. (2006). Prevalence of *in vitro* resistance to eleven standard or new antimalarial drugs among *Plasmodium falciparum* isolates from Pointe-Noire, Republic of the Congo. *Journal of Clinical Microbiology*.

[17] Eyase F. L., Akala H. M., Ingasia L., Cheruiyot A., Omondi A., Okudo C., Juma D., Yeda R., Andagalu B., Wanja E., Kamau E., Schnabel D., Bulimo W., Waters N. C., Walsh D. S., Johnson J. D. (2013). The Role of *Pfmdr1* and *Pfcrt* in changing Chloroquine, Amodiaquine, Mefloquine and Lumefantrine susceptibility in Western-Kenya *P. falciparum* samples during 2008–201. *PLoS ONE*.

[18] Mayengue P. I., Ndounga M., Davy M. M., Tandou N., Ntoumi F. (2005). In vivo chloroquine resistance and prevalence of the pfcrt codon 76 mutation in *Plasmodium falciparum* isolates from the Republic of Congo. *Acta Tropica*.

[19] de Radiguès X., Diallo K. I., Diallo M., Ngwakum P. A., Maiga H., Djimdé A., Sacko M., Doumbo O., Guthmann J.-P. (2006). Efficacy of chloroquine and sulfadoxine/pyrimethamine for the treatment of uncomplicated *falciparum* malaria in Koumantou, Mali. *Transactions of the Royal Society of Tropical Medicine and Hygiene*.

[20] Happi C. T., Gbotosho G. O., Folarin O. A., Bolaji O. M., Sowunmi A., Kyle D. E., Milhous W., Wirth D. F., Oduola A. M. J. (2006). Association between mutations in *Plasmodium falciparum* chloroquine resistance transporter and P. *falciparum* multidrug resistance 1 genes and *in vivo* amodiaquine resistance in P. *falciparum* malaria-infected children in Nigeria. *American Journal of Tropical Medicine and Hygiene*.

[21] Tinto H., Sanou B., Dujardin J.-C. (2005). Short report: usefulness of the *Plasmodium falciparum* chloroquine resistance transporter T76 genotype failure index for the estimation of *in vivo* chloroquine resistance in Burkina Faso. *The American Journal of Tropical Medicine and Hygiene*.

[22] Tinto H., Guekoun L., Zongo I., Guiguemdé R. T., D'Alessandro U., Ouédraogo J. B. (2008). Chloroquine-resistance molecular markers (Pfcrt T76 and Pfmdr-1 Y86) and amodiaquine resistance in Burkina Faso. *Tropical Medicine and International Health*.

[23] Djimdé A., Doumbo O. K., Steketee R. W., Plowe C. V. (2001). Application of a molecular marker for surveillance of chloroquine-resistant *falciparum* malaria. *The Lancet*.

[24] Djimde A. A., Barger B., Kone A., Beavogui A. H., Tekete M., Fofana B., Dara A., Maiga H., Dembele D., Toure S., Dama S., Ouologuem D., Sangare C. P. O., Dolo A., Sogoba N., Nimaga K., Kone Y., Doumbo O. K. (2010). A molecular map of chloroquine resistance in Mali. *FEMS Immunology and Medical Microbiology*.

[25] Mockenhaupt F. P., Ehrhardt S., Eggelte T. A., Agana-Nsiire P., Stollberg K., Mathieu A., Markert M., Otchwemah R. N., Bienzle U. (2005). Chloroquine-treatment failure in northern Ghana: roles of *pfcrt* T76 and *pfmdr1* Y86. *Annals of Tropical Medicine and Parasitology*.

[26] Ibrahim M. L., Gay-Andrieu F., Adehossi E., Lacroix V., Randrianarivelojosia M., Duchemin J.-B. (2007). Field-based evidence for the linkage of *pfcrt* and *pfdhfr* drug-resistant malaria genotypes and clinical profiles of severe malaria in Niger. *Microbes and Infection*.

[27] Ibrahim M. L., Hassane H., Konate L. (2008). *Plasmodium falciparum* chloroquine and pyrimethamine resistance monitoring network with molecular tools in the Niger river valley, Republic of Niger. *Bulletin de la Société de Pathologie Exotique*.

[28] Borrmann S., Binder R. K., Adegnika A. A., Missinou M. A., Issifou S., Ramharter M., Wernsdorfer W. H., Kremsner P. G. (2002). Reassessment of the resistance of *Plasmodium falciparum* to chloroquine in Gabon: implications for the validity of tests *in vitro* vs. *in vivo*. *Transactions of the Royal Society of Tropical Medicine and Hygiene*.

[29] Frank M., Lehners N., Mayengue P. I. (2011). A thirteen-year analysis of *Plasmodium falciparum* populations reveals high conservation of the mutant *pfcrt* haplotype despite the withdrawal of chloroquine from national treatment guidelines in Gabon. *Malaria Journal*.

[30] Labbo R., Czeher C., Djibrila A., Arzika I., Jeanne I., Duchemin J.-B. (2012). Longitudinal follow-up of malaria transmission dynamics in two villages in a Sahelian area of Niger during a nationwide insecticide-treated bednet distribution programme. *Medical and Veterinary Entomology*.

[31] Elissa N., Karch S., Bureau P., Ollomo B., Lawoko M., Yangari P., Ebang B., Georges A. J. (1999). Malaria transmission in a region of savanna-forest mosaic, haut-ogooué, Gabon. *Journal of the American Mosquito Control Association*.

[32] Doudou M. H., Mahamadou A., Ouba I., Lazoumar R., Boubacar B., Arzika I., Zamanka H., Ibrahim M. L., Labbo R., Maiguizo S., Girond F., Guillebaud J., Maazou A., Fandeur T. (2012). A refined estimate of the malaria burden in Niger. *Malaria Journal*.

